# No Correlation Between Plantar Flexor Muscle Volume and Sprint Performance in Sprinters

**DOI:** 10.3389/fspor.2021.671248

**Published:** 2021-09-21

**Authors:** Yuto Miyake, Tadashi Suga, Masafumi Terada, Takahiro Tanaka, Hiromasa Ueno, Yuki Kusagawa, Mitsuo Otsuka, Akinori Nagano, Tadao Isaka

**Affiliations:** ^1^Faculty of Sport and Health Science, Ritsumeikan University, Kusatsu, Japan; ^2^Graduate School of Health and Sport Science, Nippon Sport Science University, Tokyo, Japan; ^3^Japan Society for the Promotion of Science, Tokyo, Japan; ^4^Faculty of Sport Science, Nippon Sport Science University, Yokohama, Japan

**Keywords:** muscle cross-sectional area, moment arm, joint torque, stiffness, magnetic resonance imaging

## Abstract

The plantar flexor torque plays an important role in achieving superior sprint performance in sprinters. Because of the close relationship between joint torque and muscle size, a simple assumption can be made that greater plantar flexor muscles (i.e., triceps surae muscles) are related to better sprint performance. However, previous studies have reported the absence of these relationships. Furthermore, to examine these relationships, only a few studies have calculated the muscle volume (MV) of the plantar flexors. In this study, we hypothesized that the plantar flexor MVs may not be important morphological factors for sprint performance. To test our hypothesis, we examined the relationships between plantar flexor MVs and sprint performance in sprinters. Fifty-two male sprinters and 26 body size-matched male non-sprinters participated in this study. On the basis of the personal best 100 m sprint times [range, 10.21–11.90 (mean ± SD, 11.13 ± 0.42) s] in sprinters, a *K*-means cluster analysis was applied to divide them into four sprint performance level groups (*n* = 8, 8, 19, and 17 for each group), which was the optimal number of clusters determined by the silhouette coefficient. The MVs of the *gastrocnemius lateralis* (GL), *gastrocnemius medialis* (GM), and *soleus* (SOL) in participants were measured using magnetic resonance imaging. In addition to absolute MVs, the relative MVs normalized to body mass were used for the analyses. The absolute and relative MVs of the total and individual plantar flexors were significantly greater in sprinters than in non-sprinters (all *p* < 0.01, *d* = 0.64–1.39). In contrast, all the plantar flexor MV variables did not differ significantly among the four groups of sprinters (all *p* > 0.05, η^2^ = 0.02–0.07). Furthermore, all plantar flexor MV variables did not correlate significantly with personal best 100 m sprint time in sprinters (*r* = −0.253–0.002, all *p* > 0.05). These findings suggest that although the plantar flexor muscles are specifically developed in sprinters compared to untrained non-sprinters, the greater plantar flexor MVs in the sprinters may not be important morphological factors for their sprint performance.

## Introduction

Superior sprint performance is achieved through the generation of torques by muscles crossing the lower limb joints (Novacheck, 1998). Of these components, greater plantar flexor torque plays an important role in increasing ground reaction forces and shortening contact time during the stance phase while sprinting (Novacheck, 1998), which are kinetic and kinematic determinates of superior sprint performance (Morin et al., 2012). Dowson et al. (1998) reported that greater isokinetic torque of the plantar flexors was correlated with better sprint performance in athletes, including sprinters. In general, the magnitude of joint torque is mainly determined by the size of the agonist muscle group (Fukunaga et al., 2001). Indeed, previous studies have consistently reported that greater sizes of the lower limb muscles, such as hip flexors (e.g., the psoas major) and extensors (e.g., the gluteus maximus), are related to better sprint performance in sprinters (Hoshikawa et al., 2006; Sugisaki et al., 2018; Tottori et al., 2018, 2021; Miller et al., 2021). In contrast, whether the greater size of the plantar flexor muscles (i.e., the triceps surae muscles) is related to better sprint performance in sprinters remains controversial among previous studies (Kumagai et al., 2000; Kubo et al., 2011; Sugisaki et al., 2011, 2018; Tottori et al., 2018, 2021; Monte and Zamparo, 2019; Tanaka et al., 2019; Miller et al., 2021).

Using ultrasonography, Kumagai et al. (2000) reported that muscle thickness (MT) of the plantar flexors was greater in faster sprinters (<11.00 s) than in slower sprinters (≥11.0 s), defined based on their personal best 100 m sprint times. However, they did not examine the direct relationship between plantar flexor MT and sprint performance in sprinters. Monte and Zamparo (2019) reported that MTs of the *gastrocnemius lateralis* (GL), *gastrocnemius medialis* (GM), and *soleus* (SOL) were correlated with personal best 100 m sprint time in sprinters. In contrast, Kubo et al. (2011) reported that the plantar flexor MT was not correlated with personal best 100 m sprint time in sprinters. Our previous study also reported no correlations between GL and GM MTs and personal best 100 m sprint time in sprinters (Tanaka et al., 2019). Furthermore, using magnetic resonance imaging (MRI), we previously reported that the cross-sectional area (CSA) of the plantar flexors was not correlated with sprint performance (i.e., personal best 100 m sprint time and 50 m sprint velocity) in sprinters (Tottori et al., 2018, 2021). This finding corroborates the result of a study by Sugisaki et al. (2011) who reported no correlation between plantar flexor CSA and 30 m sprint time in sprinters and middle-distance runners. Nevertheless, it is well-known that compared with MT and CSA, muscle volume (MV) is a more appropriate marker to evaluate muscle size (Fukunaga et al., 2001; Akagi et al., 2009).

To the best of our knowledge, only two previous studies (Sugisaki et al., 2018; Miller et al., 2021) have examined the relationship between plantar flexor MV and sprint performance in sprinters. Sugisaki et al. (2018) reported that MVs of the gastrocnemius (GAS: i.e., a combination of the GL and GM) and SOL were not correlated with personal best 100 m sprint time in sprinters. However, they did not examine the relationship between the total MV of the three plantar flexors (i.e., the GL, GM, and SOL) and sprint performance. In addition, because the GAS can be divided into two muscles (i.e., the GL and GM), the researchers did not examine the relationship between each GAS MV and sprint performance. In a recent study, Miller et al. (2021) calculated the total and individual plantar flexor MVs in sprinters and reported negative correlations between all absolute MVs of the total and individual plantar flexors and personal best 100 m sprint time. However, these correlations were not observed after these MVs were normalized to body mass (i.e., relative MVs). Therefore, the relationship between plantar flexor size (especially MV) and sprint performance in sprinters is not fully understood. After considering comprehensively the findings from our and other studies (Sugisaki et al., 2011, 2018; Tottori et al., 2018, 2021; Tanaka et al., 2019; Miller et al., 2021), we hypothesized that the greater plantar flexor MVs may not be important morphological factors for achieving better sprint performance in sprinters.

To test our hypothesis, we calculated the total and individual MVs of the plantar flexors in sprinters to establish the relationship between plantar flexor size and sprint performance. We first compared the total and individual plantar flexor MVs between sprinters and non-sprinters to understand the level of specific development of the plantar flexor muscles in the sprinters. Second, we compared the total and individual plantar flexor MVs among four groups of sprinters, which were defined based on their personal best 100 m sprint times, to understand the impact of differing sprint performance levels on the plantar flexor MVs of sprinters. Third, we examined the relationship between the total and individual plantar flexor MVs and sprint performance in sprinters.

## Methods

### Participants

Prior to this study, we calculated *a priori* sample size using the effect sizes obtained in our previous study that examined the specific muscles (i.e., the psoas major and gluteus maximus) for superior sprint performance in sprinters (Tottori et al., 2021). To compare muscle size variable between two groups (e.g., sprinters vs. non-sprinters), the necessary minimum number of participants for each group were six, which was calculated from an effect size of 1.82 (i.e., the relative gluteus maximus CSA), α-level of 0.05, and β-level of 0.20 (80% power). In addition, to determine the relationship between muscle size variable and sprint performance in sprinters, the necessary minimum number of sprinters was 49, which was calculated from an effect size of 0.388 (i.e., the absolute psoas major CSA), α-level of 0.05, and β-level of 0.2 (80% power).

Fifty-two male sprinters (21 ± 2 years) participated in this study. All sprinters were well-trained, being involved in competitions and regular training. The best official records in a 100 m race (i.e., the personal best 100-m sprint time) within the previous 1 year in the sprinters ranged from 10.21 to 11.90 s (mean, 11.13 ± 0.42 s). The sprinters were involved in regular sprint training at least five times per week. The mean duration of training experience among the sprinters was 8.5 ± 2.4 years. In addition, 26 male non-sprinters (22 ± 1 years) whose physical characteristics (i.e., body height, body mass, and body mass index) were similar to those of the sprinters were selected as a control group (see Table 1). The body size-matched control non-sprinters were recreationally active but were not involved in any specific physical training program within the previous 3 years. Many of them had participated in recreational sports and/or physical training for 2–3 h/week. All participants were informed of the experimental procedures and provided written consent to participate in the study. This study was approved by the Ethics Committee of Ritsumeikan University (BKC-IRB-2016-047).

**Table 1:** Physical characteristics and plantar flexor muscle size variables in sprinters and non-sprinters.

	**Sprinters**	**Non-sprinters**	***P*-value**	**Cohen's *d***
	**(*n* = 52)**	**(*n* = 26)**		
Body height, cm	174.84 ± 4.96	173.64 ± 4.87	0.316	0.24
Body mass, kg	65.51 ± 5.60	65.63 ± 7.01	0.931	−0.02
Body mass index, kg/m^2^	21.40 ± 1.25	21.77 ± 2.18	0.450	−0.22
Absolute ACSA, cm^2^				
*Gastrocnemius lateralis*	11.49 ± 1.80	9.98 ± 1.76	<0.001	0.84
*Gastrocnemius medialis*	18.30 ± 3.32	15.90 ± 2.40	0.002	0.79
*Soleus*	27.24 ± 4.25	28.11 ± 3.40	0.365	−0.22
Plantar flexors	50.54 ± 7.04	46.92 ± 5.71	0.026	0.55
Relative ACSA, cm^2^/kg^2/3^				
*Gastrocnemius lateralis*	0.71 ± 0.11	0.61 ± 0.09	<0.001	0.92
*Gastrocnemius medialis*	1.13 ± 0.18	0.98 ± 0.1	<0.001	0.87
*Soleus*	1.68 ± 0.23	1.73 ± 0.21	0.228	−0.26
Plantar flexors	3.11 ± 0.37	2.89 ± 0.33	0.016	0.61
Absolute MV, cm^3^				
*Gastrocnemius lateralis*	155.24 ± 29.30	122.84 ± 24.42	<0.001	1.17
*Gastrocnemius medialis*	280.20 ± 56.22	225.74 ± 41.09	<0.001	1.05
*Soleus*	475.60 ± 81.04	428.63 ± 54.10	<0.001	0.64
Plantar flexors	911.03 ± 147.19	777.21 ± 103.67	0.008	1.00
Relative MV, cm^3^/kg				
*Gastrocnemius lateralis*	2.37 ± 0.38	1.87 ± 0.30	<0.001	1.39
*Gastrocnemius medialis*	4.27 ± 0.69	3.46 ± 0.61	<0.001	1.22
*Soleus*	7.24 ± 0.91	6.59 ± 0.95	0.004	0.71
Planter flexors	13.87 ± 1.59	11.92 ± 1.61	<0.001	1.23
Percent composition, %				
*Gastrocnemius lateralis*	17.10 ± 2.19	15.79 ± 2.30	0.004	0.59
*Gastrocnemius medialis*	30.67 ± 2.59	28.94 ± 2.43	0.006	0.68
*Soleus*	52.23 ± 3.56	55.27 ± 2.95	<0.001	−−0.90

*Values are presented as mean ± SD. ACSM, anatomical cross-sectional area; MV, muscle volume. The magnitude of the effect size (i.e., Cohen's d) is interpreted as small (0.20–0.49), medium (0.50–0.79), and large (> 0.80)*.

### MRI

To avoid any effects of changes in the muscle size secondary to heavy training during the on-season, MRI measurements in the sprinters were scheduled for the following day after the day of rest or light-intensity training during the off-season. In addition, the MRI measurements for sprinters who performed the light-intensity training were scheduled at least 12 h after this training session.

The MRI measurement was performed using a 1.5-T magnetic resonance system (Signa HDxt; GE Medical Systems, Waukesha WI, USA). To obtain images for measuring the plantar flexor MVs, we placed subjects in a supine position on the scanner bed, with both knees fully extended and both ankles set at the neutral position (i.e., 0°). Axial T_1_-weighted MRI scans of the lower leg were acquired with a standard body coil. The axial scans were obtained in successive slices with an inter distance of 10 mm with a repetition time of 600 ms, echo time of 7.6 ms, a field of view of 480 mm, and matrix size of 512 × 256 pixels.

The CSAs on each slice in the GL, GM, and SOL were measured using image analysis software (OsiriX version 5.6; OsiriX Foundation, Geneva, Switzerland). Adipose and connective tissue incursions were excluded as much as possible from each image. The maximum CSAs along muscle lengths of total and individual plantar flexors were adopted as each anatomical CSA (ACSA) (Akagi et al., 2009; Tomita et al., 2018). The MVs of the total and individual plantar flexors were calculated by summing the CSAs of all images along their length at intervals of 10 mm. To minimize the effect of the difference in body size among participants, in addition to the absolute values, the relative ACSAs and MVs of the total and individual plantar flexors normalized to body mass were used for the analysis of this study. In addition, percent MVs of individual plantar flexors to the total plantar flexors were determined to understand component characteristics within the plantar flexors.

To test the reliability for calculating the plantar flexor MVs, in this study, we repeated two times for the measurements of the total and individual plantar flexor MVs in 10 healthy young males. The coefficients of variation for the two measurements were 1.5 ± 0.9% for the total plantar flexor MV, 3.2 ± 2.4% for the GL MV, 1.7 ± 1.7% for the GM MV, and 2.2 ± 1.8% for the SOL MV. The intraclass correlation coefficients for the two measurements were 0.981 for the total plantar flexor MV, 0.940 for the GL MV, 0.979 for the GM MV, and 0.941 for the SOL MV, which can be considered as excellent reliable (Koo and Li, 2016).

### Statistical Analysis

All data are presented as mean ± SD. All data were checked for normality using the Shapiro–Wilk test. Comparisons of the measured variables between sprinters and non-sprinters were performed using independent *t*-tests. If any variables were not normally distributed, the Mann–Whitney *U*-test was used. Cohen's *d* was calculated as the effect size to determine the magnitude of difference in the measured variables between the two groups. The magnitude of this effect size was interpreted as small (0.20–0.49), medium (0.50–0.79), and large (>0.80) (Cohen, 1992).

**Figure 1:**
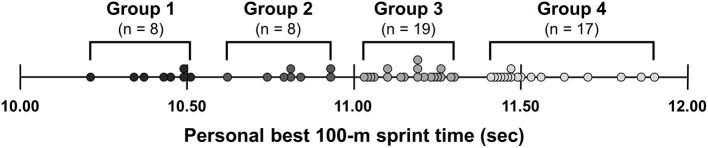
Individual plots of the personal best 100-m sprint times in sprinters. A *K*-means cluster analysis was applied to divide the sprinters into four groups [Group 1: range, 10.21–10.51 (mean, 10.41 ± 0.10) s; Group 2: range, 10.61–10.93 (mean, 10.81 ± 0.10) s; Group 3: range, 11.03–11.30 (mean, 11.17 ± 0.09) s; and Group 4: range, 11.41–11.90 (mean, 11.56 ± 0.16) s] based on their personal best 100 m sprint times.

The individual plots of the personal best 100 m sprint times in sprinters are shown in Figure 1. On the basis of the personal best 100 m sprint times, a *K*-means cluster analysis with algorithms built into the IBM SPSS software (version 19.0; IBM Corp., Armonk, NY, USA) was applied to divide the sprinters into subgroups. Because the *K*-means cluster analysis used in this study was performed with only one variable, we did not standardize the personal best 100 m sprint time to Z-score. The appropriate number of clusters was determined by the silhouette coefficient, which showed to be four clusters. Thus, the sprinters were divided into the four sprint performance level groups [Group 1 (*n* = 8): range, 10.21–10.51 (mean, 10.41 ± 0.10) s; Group 2 (*n* = 8): range, 10.61–10.93 (mean, 10.81 ± 0.10) s; Group 3 (*n* = 19): range, 11.03–11.30 (mean, 11.17 ± 0.09) s; and Group 4 (*n* = 17): range, 11.41–11.90 (mean, 11.56 ± 0.16) s]. Comparisons of the measured variables among the four groups of sprinters were performed using a one-way ANOVA, followed by pairwise comparisons using Tukey's multiple comparison procedure. If any variables were not normally distributed, the Kruskal–Wallis test was used. Eta squared (η^2^) was calculated as the effect size to determine the magnitude of difference in the measured variables between the four groups. The magnitude of this effect size was interpreted as small (0.01–0.05), medium (0.06–0.13), and large (>0.14) (Lakens, 2013).

Correlations between plantar flexor muscle size variables and personal best 100 m sprint time in sprinters were evaluated using Pearson's product-moment correlation coefficient. The magnitude of this correlation was interpreted as small (0.10–0.29), medium (0.30–0.49), and large (>0.50) (Cohen, 1992). The statistical significance was defined at *p* < 0.05. All statistical analyses were conducted using the SPSS software.

## Results

Physical characteristics and plantar flexor muscle size variables in sprinters and non-sprinters are listed in Table 1. Physical characteristics (i.e., body height, body mass, and body mass index) did not differ significantly between sprinters and non-sprinters (all *p* > 0.05, *d* = −0.22 to 0.24). The absolute and relative ACSAs of the total plantar flexors were significantly greater in sprinters than in non-sprinters (both *p* < 0.05, *d* = 0.55 and 0.61, respectively). Although the absolute and relative ACSAs of the SOL did not differ significantly between the two groups (both *p* > 0.05, *d* = −0.22 and −0.26, respectively), the absolute and relative ACSAs of the GL and GM were significantly greater in sprinters than in non-sprinters (all *p* < 0.01, *d* = 0.79–0.92). Furthermore, the absolute and relative MVs of the total plantar flexors were significantly greater in sprinters than in non-sprinters (both *p* < 0.01, *d* = 1.00 and 1.23, respectively). The absolute and relative MVs of all three plantar flexors were also significantly greater in sprinters than in non-sprinters (all *p* < 0.01, *d* = 0.64–1.39). In addition, percent MVs of the GM and GL to the total plantar flexors were significantly higher in sprinters than in non-sprinters (both *p* < 0.01, *d* = 0.59 and 0.68, respectively). In contrast, a percent MV of the SOL to the total plantar flexors was significantly lower in sprinters than in non-sprinters (*p* < 0.001, *d* = −0.90).

Physical characteristics and plantar flexor muscle size variables in four sprint performance level groups of sprinters are listed in Table 2. Body height and body mass did not differ significantly among the four groups of sprinters (both *p* > 0.05, η^2^ = 0.04 and 0.12). In contrast, one-way ANOVA for body mass index revealed significant differences among the four groups (*p* = 0.019, η^2^ = 0.19). *Post-hoc* pairwise comparisons indicated that the body mass index was significantly higher in Groups 1 and 2 than in Group 4 (both *p* < 0.05). All absolute and relative ACSAs and MVs of the total and individual plantar flexors did not differ significantly among the four groups (all *p* > 0.05, η^2^ = 0.02–0.12). Furthermore, percent MVs of each muscle to the total plantar flexors also did not differ significantly among the four groups (all *p* > 0.05, η^2^ = 0.01–0.02).

**Table 2:** Physical characteristics and plantar flexor muscle size variables in four sprint performance level groups of sprinters based on their personal best 100-m sprint times.

	**Group 1 (*n* = 8)**	**Group 2 (*n* = 8)**	**Group 3 (*n* = 19)**	**Group 4 (*n* = 17)**	***P*-value**	**η^**2**^**
Body height, cm	176.75 ± 4.98	175.55 ± 6.05	173.86 ± 4.10	174.70 ± 5.42	0.562	0.04
Body mass, kg	68.58 ± 2.92	67.04 ± 6.86	65.68 ± 5.50	63.14 ± 5.45	0.105	0.12
Body mass index, kg/m^2^	21.99 ± 1.39	21.70 ± 1.11	21.70 ± 1.24	20.65 ± 0.99^*^^†^	0.019	0.19
Absolute ACSA, cm^2^						
*Gastrocnemius lateralis*	11.45 ± 1.82	10.91 ± 2.08	12.22 ± 1.77	10.96 ± 1.55	0.145	0.11
*Gastrocnemius medialis*	19.04 ± 3.62	17.47 ± 3.97	18.96 ± 3.77	17.62 ± 2.20	0.538	0.05
*Soleus*	28.27 ± 4.67	27.23 ± 5.17	27.75 ± 3.53	26.19 ± 4.50	0.633	0.04
Plantar flexors	51.42 ± 8.32	50.31 ± 9.24	52.12 ± 6.71	48.47 ± 5.63	0.474	0.05
Relative ACSA, cm^2^/kg^2/3^						
*Gastrocnemius lateralis*	0.68 ± 0.10	0.66 ± 0.11	0.75 ± 0.11	0.69 ± 0.10	0.074	0.12
*Gastrocnemius medialis*	1.14 ± 0.21	1.05 ± 0.20	1.16 ± 0.21	1.11 ± 0.11	0.697	0.04
*Soleus*	1.69 ± 0.28	1.65 ± 0.29	1.70 ± 0.18	1.65 ± 0.25	0.853	0.02
Plantar flexors	3.07 ± 0.47	3.04 ± 0.45	3.20 ± 0.35	3.06 ± 0.29	0.519	0.04
Absolute MV, cm^3^						
*Gastrocnemius lateralis*	163.76 ± 29.49	150.14 ± 38.08	161.76 ± 31.13	146.33 ± 21.14	0.337	0.07
*Gastrocnemius medialis*	299.46 ± 43.26	280.04 ± 80.57	285.84 ± 80.57	263.02 ± 42.85	0.442	0.05
*Soleus*	514.21 ± 94.60	471.87 ± 92.83	477.50 ± 73.37	457.05 ± 77.92	0.445	0.05
Planter flexors	977.42 ± 153.41	906.04 ± 191.56	925.10 ± 148.21	866.41 ± 115.86	0.345	0.07
Relative MV, cm^3^/kg						
*Gastrocnemius lateralis*	2.38 ± 0.36	2.22 ± 0.41	2.46 ± 0.42	2.32 ± 0.31	0.337	0.05
*Gastrocnemius medialis*	4.36 ± 0.57	4.20 ± 0.89	4.34 ± 0.78	4.17 ± 0.55	0.442	0.02
*Soleus*	7.49 ± 1.28	7.01 ± 0.98	7.25 ± 0.77	7.23 ± 0.89	0.445	0.02
Planter flexors	14.24 ± 1.98	13.42 ± 1.82	14.05 ± 1.71	13.72 ± 1.17	0.345	0.03
Percent composition, %						
*Gastrocnemius lateralis*	16.76 ± 1.58	16.69 ± 3.31	17.50 ± 1.93	16.99 ± 2.20	0.774	0.02
*Gastrocnemius medialis*	30.77 ± 2.28	31.06 ± 2.93	30.73 ± 2.54	30.38 ± 2.82	0.945	0.01
*Soleus*	52.47 ± 3.43	52.25 ± 3.75	51.76 ± 3.32	52.63 ± 4.02	0.911	0.01

*Values are presented as mean ± SD. η^2^; eta squared, ACSM; anatomical cross-sectional area, MV, muscle volume. The magnitude of the effect size (i.e., η^2^) is interpreted as small (0.01–0.05), medium (0.06–0.13), and large (>0.14)*.

* *Significant difference (P = 0.046) from Group 1*.

† *Significant difference (P = 0.044) from Group 3*.

The Pearson's product-moment correlation coefficients between plantar flexor muscle variables and personal best 100 m sprint time in sprinters are shown in Table 3. All absolute and relative ACSAs and MVs of the total and individual plantar flexors did not correlate significantly with personal best 100 m sprint time in sprinters (*r* = −0.249 to 0.101, all *p* > 0.05). In addition, percent MVs of individual plantar flexors to the total plantar flexors did not correlate significantly with personal best 100 m sprint time in sprinters (*r* = −0.119 to 0.113, all *p* > 0.05).

**Table 3:** Pearson's product moment correlation coefficients between plantar flexor muscle size variables and personal best 100-m race time in sprinters.

	**Sprinters (** ***n*** **=** **52)**	
	***r***	***P-*value**
Absolute ACSA		
*Gastrocnemius lateralis*	−0.049	0.730
*Gastrocnemius medialis*	−0.144	0.308
*Soleus*	−0.177	0.209
Plantar flexors	−0.154	0.277
Relative ACSA		
*Gastrocnemius lateralis*	0.101	0.475
*Gastrocnemius medialis*	−0.100	0.483
*Soleus*	−0.047	0.743
Plantar flexors	0.011	0.937
Absolute MV		
*Gastrocnemius lateralis*	−0.134	0.343
*Gastrocnemius medialis*	−0.249	0.075
*Soleus*	−0.226	0.106
Plantar flexors	−0.247	0.078
Relative MV		
*Gastrocnemius lateralis*	0.030	0.832
*Gastrocnemius medialis*	−0.133	0.346
*Soleus*	−0.072	0.610
Planter flexors	−0.096	0.498
Percent composition		
*Gastrocnemius lateralis*	0.113	0.426
*Gastrocnemius medialis*	−0.119	0.403
*Soleus*	0.019	0.895

*ACSM, anatomical cross-sectional area; MV, muscle volume. The magnitude of the correlation is interpreted as small (0.10–0.29), medium (0.30–0.49), and large (>0.50)*.

## Discussion

Prior to this study, the relationship between plantar flexor size and sprint performance in sprinters was controversial among previous studies (Kumagai et al., 2000; Kubo et al., 2011; Sugisaki et al., 2011, 2018; Tottori et al., 2018, 2021; Monte and Zamparo, 2019; Tanaka et al., 2019; Miller et al., 2021). In addition, despite the fact that MV is a more appropriate marker for evaluating muscle size (Fukunaga et al., 2001; Akagi et al., 2009), only two previous studies (Sugisaki et al., 2018; Miller et al., 2021) have examined the relationships between plantar flexor MVs and sprint performance in sprinters. Miller et al. (2021) reported that although negative correlations were observed between absolute MVs of the total and individual plantar flexors and personal best 100 m sprint time in sprinters, all the relative MVs normalized to body mass were not correlated with the sprint performance. Moreover, Sugisaki et al. (2018) reported the absence of correlations between absolute and relative MVs of the GAS and SOL and personal best 100 m sprint time in sprinters. In this study, we observed that absolute and relative MVs of the total and individual plantar flexors did not differ among the four sprint performance level groups of sprinters. Furthermore, all the plantar flexor MV variables were not correlated with personal best 100 m sprint time in sprinters. The results of the present study corroborate those of previous studies (Sugisaki et al., 2018; Miller et al., 2021). Therefore, the findings of the present and previous studies suggest that the plantar flexor MVs may not be important morphological factors for achieving superior sprint performance in sprinters.

We and others have previously reported that the total plantar flexor CSA, which measured at proximal 30% of the lower leg length, was not correlated with sprint performance (i.e., personal best 100 m sprint time, 50 m sprint velocity, and 30 m sprint time) in sprinters (Sugisaki et al., 2011; Tottori et al., 2018, 2021). In this study, we calculated the ACSAs, which are defined as the maximum CSAs along each muscle length (Akagi et al., 2009; Tomita et al., 2018), of the total and individual plantar flexors, because no previous study has examined the relationships between the total and individual plantar flexor ACSAs and sprint performance in sprinters. We found that absolute and relative ACSAs of the total and individual plantar flexors did not differ among the four sprint performance level groups of sprinters. Furthermore, all the plantar flexor ACSA variables were not correlated with personal best 100 m sprint time in sprinters. Therefore, these findings support the absence of the relationships between plantar flexor MVs and sprint performance obtained in the present and previous studies (Sugisaki et al., 2018; Miller et al., 2021).

Dowson et al. (1998) reported that isokinetic plantar flexor torque was correlated with sprint performance (i.e., sprint velocities at acceleration and maximal sprinting phases) in athletes. The magnitude of maximal torque of several joints is determined not only by muscle size but also by joint moment arm (MA) dimension (Blazevich et al., 2009; Baxter and Piazza, 2014; Hori et al., 2020; Tottori et al., 2020). In a previous study, Baxter and Piazza (2014) reported a positive correlation between MA dimension and isokinetic torque of the plantar flexors in untrained individuals. In addition, our previous study determined that despite there was no correlation between quadriceps femoris CSA and sprint performance (i.e., personal best 100 m sprint time and 50 m sprint velocity) in sprinters, greater knee extensor MA was correlated with better sprint performance (Miyake et al., 2017). Although no study has examined the relationship between plantar flexor MA and sprint performance, the MA dimension, rather than muscle size, of the plantar flexors in sprinters may be a more important morphological factor for achieving superior sprint performance, potentially by enhancing the plantar flexor torque during sprinting.

Kumagai et al. (2000) reported that the fascicle lengths of the plantar flexors (i.e., the GL and GM) were longer in faster sprinters (personal best 100 m sprint time for <11.00 s) than in slower sprinters (personal best 100 m sprint time for ≥11.00 s). Furthermore, previous studies reported that longer fascicles of the GL and GM were correlated with better personal best 100 m sprint time in sprinters (Kumagai et al., 2000; Abe et al., 2001; Monte and Zamparo, 2019). This may be because longer fascicles of the lower limb muscles contribute to achieving higher muscle contractile speeds during sprinting (Kumagai et al., 2000; Abe et al., 2001). In addition, Drazan et al. (2019) reported that longer fascicle of the GM was correlated with higher isokinetic torques of the plantar flexors in untrained individuals, with a larger correlation for faster contraction than slower contraction. Therefore, in addition to the joint MA dimension, the fascicle length, rather than the muscle size, of the plantar flexors may be a more important morphological factor for achieving superior sprint performance in sprinters, potentially by enhancing muscle contractile speeds and joint torques of the plantar flexors during sprinting.

Our previous study reported that higher joint stiffness (calculated from the slope of the linear portion of the torque–angle curve during passive dorsiflexion) of the plantar flexors was correlated with better personal best 100 m sprint time in sprinters (Takahashi et al., 2018). The magnitude of the plantar flexor joint stiffness is mainly determined by the agonist muscle size (Suga et al., 2021). Based on these findings, it can be assumed that greater plantar flexor muscles may be related to better sprint performance due to increased plantar flexor joint stiffness. Nevertheless, the present findings may refute this possibility. In a previous study, Miyamoto et al. (2019) reported that higher stiffness of the knee extensor muscle (i.e., vastus lateralis) measured using ultrasound elastography was correlated with better personal best 100 m sprint time in sprinters; however, no study has examined the relationship between the ultrasound elastography-measured plantar flexor muscle stiffness and sprint performance. In addition, Ando and Suzuki (2019) reported that stiffer plantar flexors may be effective in archiving higher plantar flexor torque production. Therefore, clarifying the relationship between plantar flexor muscle stiffness and sprint performance in sprinters may contribute to our understanding of the present findings.

This study determined that most absolute and relative ACSAs and MVs of the total and individual plantar flexors, excluding absolute and relative ACSAs of the SOL, were greater in sprinters than in body size-matched non-sprinters. The results of the present study corroborate with those of previous studies that compared the plantar flexor muscle size between sprinters and non-sprinters (Abe et al., 2000; Kubo et al., 2011 Handsfield et al., 2017; Fukutani et al., 2020; Miller et al., 2021; Tottori et al., 2021). In addition, the present study determined that percent MVs of the GM and GL to the total plantar flexors were higher in sprinters than in non-sprinters, whereas a percent MV of the SOL to the total plantar flexors was lower in sprinters than in non-sprinters. These findings are consistent with the results of our previous study (Fukutani et al., 2020). Thus, our findings suggest that compared to untrained non-sprinters, sprinters have plantar flexor muscles with a higher composition of the GL and GM relative to that of the SOL. Despite this unique plantar flexor feature in the sprinters, the present study found that the percent MVs of each plantar flexor muscle were not correlated with personal best 100 m sprint time. Therefore, in addition to the muscle size, the muscle distribution of the plantar flexors may not be an important morphological factor for achieving superior sprint performance in sprinters.

The present study had some limitations. First, we did not measure 100 m sprint times in non-sprinters. This measurement may contribute to our understanding of the degree of differences in the sprint performance between sprinters and non-sprinters. We also did not measure isokinetic plantar flexor torque in participants. In particular, because the isokinetic plantar flexor torque may be an important functional factor for sprint performance (Dowson et al., 1998), this measurement may help interpret the difference in sprint performance among sprinters. Moreover, we did not measure body composition in participants. In general, body fat percentage and whole-body fat mass are lower in sprinters than in untrained non-sprinters (Abe et al., 2001; Aikawa et al., 2020). In addition, a series of studies by Abe et al. (2001, 2019) reported that lower body fat percentage and whole-body fat mass were correlated with better personal best 100 m sprint time in sprinters. Considering these findings, the whole-body muscle mass, rather than the body mass, might be appropriate as a variable for normalizing the plantar flexor size variables. Furthermore, we did not survey detailed information of training status that contributes to the development of the plantar flexor muscles in sprinters. Previous studies have reported that eccentric resistance training is effective for increasing the fascicle length of the lower limb muscles (Blazevich et al., 2007; Baroni et al., 2013; Timmins et al., 2016), including the plantar flexor muscles (Crill et al., 2014; Geremia et al., 2019), which are important morphological factors for sprint performance (Kumagai et al., 2000; Abe et al., 2001; Monte and Zamparo, 2019). Therefore, the lacks of these measurements and questionnaire data are major limitations of this study.

Second, we examined only the relationship between plantar flexor muscle size and sprint performance in sprinters. However, interactions among the sizes of the lower limb muscles (e.g., the size ratio) may play important roles in achieving superior sprint performance in sprinters (Hoshikawa et al., 2006; Sugisaki et al., 2018). Of these interactions, a greater ratio of the quadriceps femoris size relative to the plantar flexor size may be related to sprint performance in sprinters because it appears to be useful in lowering the moment of inertia of the legs during the swinging phase. To examine this possibility, we conducted an additional analysis using our previous data (Tottori et al., 2021). This result showed that the ratio of the quadriceps femoris CSA relative to the plantar flexor CSA did not correlate with personal best 100 m sprint time in sprinters (*r* = −0.122, *p* = 0.369). Nevertheless, to clarify this result, further studies are needed to determine the effect of interactions among the plantar flexor MVs and other lower limb MVs, especially the quadriceps femoris MVs, on sprint performance in sprinters.

Third, we used only the personal best 100 m sprint time, as a sprint performance parameter. This parameter inevitably includes various phases during 100 m sprinting (Otsuka et al., 2015; Miyake et al., 2017). Monte and Zamparo (2019) reported that MTs of the three plantar flexors were correlated with sprint velocity during a 20 m sprint in sprinters. Considering their findings, the plantar flexor size may contribute to achieving superior performance during the block start and/or acceleration phases. The plantar flexor torque may play an important role for block clearance performance more than other lower limb joint torques (Brazil et al., 2017; Sado et al., 2020), which may be related to accelerating sprint velocity during the initial phase while a 100 m sprinting (Macadam et al., 2019; Nagahara et al., 2020; Sado et al., 2020). Furthermore, the plantar flexor torque may be the greatest source of positive work during the stance phase when maximum acceleration sprinting among all joint torques of the lower limb (Schache et al., 2019). Further studies are needed to determine the relationships of the plantar flexor MVs with biomechanical variables, especially plantar flexor torque, during the specific phases of the 100 m sprint in sprinters.

Last, we performed MRI measurements in sprinters during the off-season to avoid any effects of changes in the muscle size secondary to heavy training during the on-season. Thus, there was a lag between the time when measured MRI and time when recorded personal best 100-m sprint time in sprinters. To accurately determine the relationships between plantar flexor size and sprint performance, further studies are needed to follow this relationship with periodic MRI measurements for sprinters throughout a season.

In conclusion, this study determined that although the plantar flexor muscles are specifically developed in sprinters compared to untrained non-sprinters, the greater plantar flexor MVs in the sprinters may not be important morphological factors for achieving their superior sprint performance.

## Data Availability Statement

The raw data supporting the conclusions of this article will be made available by the authors, without undue reservation.

## Ethics Statement

The studies involving human participants were reviewed and approved by the Ethics Committee of Ritsumeikan University. The patients/participants provided their written informed consent to participate in this study.

## Author Contributions

YM and TS conceived and designed the experiment, analyzed data, and wrote the manuscript. YM, TS, MT, TT, HU, YK, and MO performed experiments. YM, TS, MT, TT, HU, YK, MO, AN, and TI interpreted results of experiments. TS, MT, MO, AN, and TI edited and revised the manuscript. All authors have read and approved the manuscript.

## Funding

This study was supported by Grant-in-Aid for Scientific Research from the Japanese Ministry of Education, Culture, Sports, Science and Technology (#15K16497 to TS and #15H03077 to TI) and the Center of Innovation Program from Japan Science and Technology Agency (#JPMJCE1306 to TS and TI).

## Conflict of Interest

The authors declare that the research was conducted in the absence of any commercial or financial relationships that could be construed as a potential conflict of interest.

## Publisher's Note

All claims expressed in this article are solely those of the authors and do not necessarily represent those of their affiliated organizations, or those of the publisher, the editors and the reviewers. Any product that may be evaluated in this article, or claim that may be made by its manufacturer, is not guaranteed or endorsed by the publisher.
